# Echinocandin Resistance in Candida Species Isolates from Liver Transplant Recipients

**DOI:** 10.1128/AAC.01229-16

**Published:** 2017-01-24

**Authors:** Gwénolé Prigent, Nawel Aït-Ammar, Eric Levesque, Arnaud Fekkar, Jean-Marc Costa, Sarra El Anbassi, Françoise Foulet, Christophe Duvoux, Jean-Claude Merle, Eric Dannaoui, Françoise Botterel

**Affiliations:** a Unité de Parasitologie-Mycologie, Département de Virologie, Bactériologie-Hygiène, Parasitologie-Mycologie, DHU VIC, CHU Henri Mondor, AP-HP, Créteil, France; b EA Dynamyc UPEC, ENVA, Faculté de Médecine, Créteil, France; c Réanimation Digestive et Hépato-Biliaire, Service d'Anesthésie et des Réanimations Chirurgicales, CHU Henri Mondor, AP-HP, Créteil, France; d Service de Parasitologie-Mycologie, CHU La Pitié-Salpêtrière, AP-HP, Paris, France; e Laboratoire Cerba, Saint-Ouen-l'Aumône, France; f Service d'Hépatologie, CHU Henri Mondor, AP-HP, Créteil, France; g Unité de Parasitologie-Mycologie, Service de Microbiologie, Université Paris-Descartes, Faculté de Médecine, Hôpital Européen Georges Pompidou, AP-HP, Paris, France

**Keywords:** Candida, Candida albicans, Candida glabrata, Candida dubliniensis, echinocandin, liver transplantation, resistance

## Abstract

Liver transplant recipients are at risk of invasive fungal infections, especially candidiasis. Echinocandin is recommended as prophylactic treatment but is increasingly associated with resistance. Our aim was to assess echinocandin drug resistance in Candida spp. isolated from liver transplant recipients treated with this antifungal class. For this, all liver-transplanted patients in a University Hospital (Créteil, France) between January and June of 2013 and 2015 were included. Susceptibilities of Candida isolates to echinocandins were tested by Etest and the EUCAST reference method. Isolates were analyzed by *FKS* sequencing and genotyped based on microsatellites or multilocus sequence typing (MLST) profiles. Ninety-four patients were included, and 39 patients were colonized or infected and treated with echinocandin. Echinocandin resistance appeared in 3 (8%) of the treated patients within 1 month of treatment. One patient was colonized by resistant Candida glabrata, one by resistant Candida dubliniensis, and one by resistant Candida albicans. Molecular analysis found three mutations in *FKS2* HS1 (F659S, S663A, and D666E) for C. glabrata and one mutation in *FKS1* HS1 (S645P) for C. dubliniensis and C. albicans. Susceptible and resistant isolates belonged to the same genotype. To our knowledge, this is the first study on echinocandin resistance in Candida spp. in a liver transplant population. Most resistant isolates were found around/in digestive sites, perhaps due to lower diffusion of echinocandin in these sites. This work documents the risk of emergence of resistance to echinocandin, even after short-term treatment.

## INTRODUCTION

Patients undergoing liver transplantation (LT) are specifically at risk of developing invasive fungal infection (IFI). It has been shown that IFI occurs early after LT (≤2 months) (1). Invasive candidiasis (IC) is the most common post-LT IFI, with Candida albicans (50 to 60%) and Candida glabrata (about 20%) the main species responsible (2).

Antifungal prophylaxis is now a standard intervention for liver transplant recipients. After using fluconazole or liposomal amphotericin B for several years, echinocandin is now the new recommended treatment in patients with major risk factors for at least 2 to 4 weeks or until resolution of the risk factors (3). Echinocandin drugs, which inhibit the synthesis of beta-1,3 glucan in the fungal cell wall, are attractive, thanks to their good *in vitro* activity against Candida spp. (4, 5), excellent safety profile, and favorable pharmacokinetics (6). However, their expanding use can promote the emergence of resistance in Candida spp., especially among patients receiving long-term therapy (7, 8). The molecular mechanisms underlying the acquired clinical resistance include point mutations within hot spot (HS) regions of *FKS* genes encoding subunits of glucan synthase (9, 10). These mutations are an important risk factor for therapy failure (11). However, it has been reported that the development of such resistance is directly linked to prior exposure (7). In the literature, mutations inducing C. albicans resistance are most commonly found on the *FKS1* gene, and especially at amino acids 641 to 649 and amino acids 1345 to 1365 in HS1 and HS2, respectively. The substitutions concerning Ser-645 (S645P/F/Y) and Phe-641 (F641S/L) are the most frequent and are responsible for the most pronounced phenotypes (12–14). Mutations in C. glabrata are most commonly found on the *FKS2* gene (15), with the most frequent substitutions at Ser-663 (S663F/P) and Phe-659 (F659S/V/Y) (8). Echinocandin resistance has also been described in other Candida species, especially in Candida krusei and Candida tropicalis (16, 17). Only two case reports on echinocandin resistance in liver transplant recipient populations are available (7, 18). In this work, our aim was to assess echinocandin drug resistance in Candida spp. developed in liver transplant recipients after the initiation of echinocandin treatment.

## RESULTS

### Patients included.

A total of 94 liver transplant recipients were enrolled during the first 6 months of 2013 (*n* = 52) and during the same period in 2015 (*n* = 42). The median age of the recipients was 54.5 (range, 22 to 70) years. Most of them (77%) were men. The two most common etiologies for LT were cirrhosis (50%) and hepatocellular carcinoma (33%). Among these 94 patients, 56 (60%) received antifungal treatment, which included caspofungin (*n* = 41 [73%]), micafungin (*n* = 2 [4%]), and fluconazole (*n* = 13 [23%]). Of the patients treated with echinocandin, 39 [91%] were colonized and/or developed an IC.

### Echinocandin susceptibility test results.

Echinocandin resistance was detected in 3 (8%) of the treated patients. The isolates revealed one patient with resistant C. glabrata (P1), one with resistant C. dubliniensis (P2), and one with resistant C. albicans (P3). The echinocandin treatment was prophylactic for P1 and P3 and curative for P2. The echinocandin MICs of all the isolates from these patients were tested using Etest for all echinocandins and the EUCAST reference method for only anidulafungin and micafungin (Table 1). In the case of P1, 24 resistant C. glabrata isolates were recovered from the urine (*n* = 11), anus (*n* = 8), and inguinal fold (*n* = 5) (Table 2). The first resistant isolates appeared in the anus and the urine 14 days after initiation of caspofungin treatment. High MICs were then detected in the inguinal fold but not in other sites. For P2, five resistant C. dubliniensis isolates were recovered from the abdominal fluids (*n* = 4) and mouth (*n* = 1). The first resistant isolate was detected in the mouth 27 days after the beginning of caspofungin treatment. Moreover, the patient had had prior caspofungin exposure for 23 days 3 months previously to treat IC (Table 3). For P3, the only resistant isolate was recovered in the anus, though the patient was not on caspofungin treatment at the time but had received 24-day prophylactic treatment of caspofungin, which was stopped a week earlier.

**TABLE 1:** MIC values, as determined by EUCAST and Etest, and *FKS* sequencing results of studied strains

Time/source^*a*^	MIC (mg/liter) result for antifungal tested^*b*^					*FKS* genotype^*c*^
	ANF		MICA		CAS (Etest)	
	Etest	EUCAST	Etest	EUCAST		
Patient 1; C. glabrata						
D−2/mouth	0.012	0.062	0.023	0.031	0.125	Not performed
D−2/nose	0.012	0.062	0.016	0.031	0.125	Not performed
D−2/inguinal	0.012	0.031	0.016	0.031	0.125	Wild type
D−2/anus	0.016	0.031	0.016	0.015	0.125	Wild type
D−2/urine	0.012	0.062	0.016	0.015	0.125	Wild type
D−2/BAL	0.012	0.062	0.023	0.015	0.125	Not performed
D6/mouth	0.012	0.062	0.023	0.015	0.125	Not performed
D6/nose	0.012	0.062	0.023	0.015	0.125	Not performed
D6/axillary	0.012	0.031	0.023	0.015	0.125	Not performed
D6/anus	0.012	0.062	0.016	0.015	0.125	Not performed
D6/urine	0.016	0.062	0.016	0.015	0.125	Not performed
D12/mouth	0.012	0.062	0.016	0.015	0.125	Not performed
D12/nose	0.012	0.062	0.016	0.015	0.125	Not performed
D12/axillary	0.012	0.062	0.016	0.015	0.125	Not performed
D12/anus	0.047	0.062	0.064	0.031	0.38 (I)	*FKS2*-F659S
D12/urine	0.125	0.125 (R)	0.064	0.062 (R)	0.38 (I)	*FKS2*-F659S
D19/mouth	0.016	0.031	0.016	0.015	0.125	Not performed
D19/axillary	0.012	0.031	0.016	0.015	0.125	Not performed
D19/anus	0.012	0.031	0.023	0.015	0.125	Not performed
D19/urine	0.25 (I)	0.125 (R)	0.064	0.062 (R)	0.38 (I)	*FKS2*-F659S
D26/inguinal	0.19 (I)	0.125 (R)	0.064	0.062 (R)	0.38 (I)	*FKS2*-F659S
D26/anus	0,19 (I)	0.125 (R)	0.064	0.015	0.38 (I)	*FKS2*-F659S
D26/urine	0.064	0.125 (R)	0.064	0.062 (R)	0.38 (I)	*FKS2*-F659S
D33/urine	0.125	0.125 (R)	0.094 (I)	0.062 (R)	0.38 (I)	*FKS2*-F659S
D40/mouth	0.016	0.031	0.016	0.015	0.125	Not performed
D40/inguinal	0.19 (I)	0.125 (R)	0.047	0.062 (R)	0.38 (I)	*FKS2*-F659S
D40/anus	0.125	0.125 (R)	0.047	0.062 (R)	0.38 (I)	*FKS2*-F659S
D40/urine	0.25 (I)	0.125 (R)	0.064	0.062 (R)	0.38 (I)	*FKS2*-F659S
D40/BAL	0.016	0.031	0.016	0.015	0.125	Not performed
D47/anus	0.012	0.062	0.016	0.015	0.38 (I)	Wild-type
D47/urine	0.064	0.25 (R)	0.047	0.062 (R)	0.38 (I)	*FKS2*-F659S
D54/mouth	0.012	0.031	0.016	0.015	0.125	Not performed
D54/inguinal	0.125	0.125 (R)	0.047	0.031	0.38 (I)	*FKS2*-F659S
D54/anus	0.012	0.062	0.016	0.125 (R)	0.125	Wild-type
D54/urine	0.032	0.125 (R)	0.047	0.062 (R)	0.38 (I)	*FKS2*-F659S
D61/anus	0.25 (I)	0.125 (R)	0.25 (R)	0.125 (R)	0.38 (I)	*FKS2*-F659S
D61/urine	0.25 (I)	0.5 (R)	0.25 (R)	0.25 (R)	0.75 (R)	*FKS2*-F659S/*FKS2*-S663A
D68/urine	0.38 (I)	1 (R)	0.38 (R)	0.25 (R)	0.75 (R)	*FKS2*-F659S/*FKS2*-S663A
D75/mouth	0.016	0.062	0.023	0.015	0.125	Not performed
D75/inguinal	0.38 (I)	2 (R)	0.25 (R)	0.5 (R)	0.38 (I)	*FKS2*-F659S/*FKS2*-S663A
D75/anus	0.25 (I)	0.5 (R)	0.25 (R)	0.5 (R)	0.5 (R)	*FKS2*-F659S/*FKS2*-S663A
D75/urine	0.25 (I)	0.5 (R)	0.25 (R)	0.25 (R)	0.5 (R)	*FKS2*-F659S/*FKS2*-S663A
D82/mouth	0.012	0.062	0.016	0.015	0.125	Not performed
D82/urine	0.25 (I)	1 (R)	0.19 (I)	0.5 (R)	0.5 (R)	*FKS2*-F659S/*FKS2*-S663A
D89/inguinal	0.38 (I)	1 (R)	0.25 (R)	0.5 (R)	0.38 (I)	*FKS2*-F659S/*FKS2*-S663A
D89/anus	0.38 (I)	1 (R)	0.25 (R)	8 (R)	4 (R)	*FKS2*-F659S/*FKS2*-S663A/*FKS2*-D666E
D96/axillary	0.016	0.031	0.016	0.015	0.125	Not performed
Patient 2; C. dubliniensis						
D15/mouth	0.006	0.015	0.016	0.015	0.064	Wild type
D43/anus	0.004	0.015	0.032	0.015	0.064	Wild type
D43/bile	0.008	0.015	0.023	0.015	0.064	Wild-type
D113/mouth	0.19	0.5 (R)	0.38 (I)	0.5 (R)	1 (R)	*FKS1*-S645P
D113/drainage fluid	0.25	0.5 (R)	0.25	0.5 (R)	1.5 (R)	*FKS1*-S645P
D120/mouth	0.008	0.015	0.047	0.015	0.125	Wild type
D120/abscess	0.25	0.125 (R)	0.38 (I)	0.5 (R)	3 (R)	*FKS1*-S645P
D120/abdominal collection	0.25	0.5 (R)	0.38 (I)	0.5 (R)	1 (R)	*FKS1*-S645P
D127/peritoneal fluid	0.25	0.125 (R)	0.25	0.5 (R)	1.5 (R)	*FKS1*-S645P
Patient 3; C. albicans						
D0/inguinal	0.016	0.015	0.004	0.015	0.125	Wild type
D10/mouth	0.008	0.015	0.064	0.015	0.125	Not performed
D18/mouth	0.016	0.015	0.002	0.015	0.125	Not performed
D32/anus	0.5 (I)	0.125 (R)	0.5 (I)	1 (R)	0.38 (I)	*FKS1*-S645P

a D0 corresponds to the day of liver transplantation.

b ANF, anidulafungin; MICA, micafungin; R, resistant; I, intermediate; BAL, bronchoalveolar lavage. For the EUCAST broth microdilution method, isolate categorizations were performed according to the EUCAST breakpoints. For the Etest method, isolate categorizations were performed according to the manufacturer's instructions (for *C*. albicans and C. dubliniensis, S ≤ 0.25, I = 0.38 to 0.75, and R ≥ 1; for C. glabrata, S ≤ 0.125, I = 0.19 to 0.38, and R ≥ 0.5; for anidulafungin and micafungin, S ≤ 0.06, I = 0.094 to 0.19, and R ≥ 0.25). Only the resistant and intermediate isolates are marked in parentheses.

c For C. glabrata, *FKS1* HS1, *FKS1* HS2, *FKS2* HS1, and *FKS2* HS2 sequencing was performed. For C. albicans and C. dubliniensis, *FKS1* HS1 and *FKS1* HS2 sequencing was performed.

**TABLE 2:** C. glabrata isolates from P1

Day^*a*^ of surveillance	Treatment^*b*^	Fungal surveillance culture^*c*^					Other^*c*^	
		Mouth	Nose	Axillary	Inguinal	Anus	Urine	BAL
D−2	CAS D0 to D96	S	S	−	S	S	S	S
D6		S	S	S	−	S	S	
D12		S	S	S	−	R	R	
D19		S	−	S	−	S	R	
D26		−	−	−	R	R	R	
D33		−	−	−	−	−	R	
D40		S	−	−	R	R	R	S
D47		−	−	−	−	R	R	
D54		S	−	−	R	R	R	
D61		−	−	−	−	R	R	
D68		−	−	−	−	−	R	
D75		S	−	−	R	R	R	
D82		S	−	−	−	−	R	
D89		−	−	−	R	R	−	
D96		−	−	S	−	−	−	

a D0 is considered the day of liver transplantation.

b CAS, caspofungin.

c S, isolate susceptible to echinocandins using the Etest and EUCAST reference method; R, isolate resistant or intermediate to echinocandins using the Etest and EUCAST reference method; −, negative culture or unavailable isolate; BAL, bronchoalveolar lavage (fluid).

**TABLE 3:** C. dubliniensis isolates from P2

Day^*a*^ of surveillance	Treatment^*b*^	Fungal surveillance culture^*c*^					Other^*c*^	
		Mouth	Nose	Axillary	Inguinal	Anus	Urine	Other
D1	CAS D12 to D35	−	−	−	−	−	−	
D8		−	−	−	−	−	−	
D15		S	−	−	−	−	−	
D22		−	−	−	−	−	−	
D43 (hospital discharge to D86)		−	−	−	−	S	−	S (bile)
D86	CAS D86 to D122	−	−	−	−	−	−	
D113		R	−	−	−	−	−	R (drain)
D120		S	−	−	−	−	−	R (abdominal abscess)
D127	AMB D122 to D131	−	−	−	−	−	−	R (peritoneal fluid)
D134	VOR D131 to D186	−	−	−	−	−	−	
D154		−	−	−	−	−	−	
D162		−	−	−	−	−	−	
D169		−	−	−	−	−	−	
D176		−	−	−	−	−	−	
D183		−	−	−	−	−	−	

a D0 is considered the day of liver transplantation.

b CAS, caspofungin; AMB, liposomal amphotericin B; VOR, voriconazole.

c S, isolate susceptible to anidulafungin and micafungin using the EUCAST reference method; R, isolate resistant to anidulafungin and micafungin using the EUCAST reference method; −, negative culture or unavailable isolate.

### Identification of mutations in *FKS* genes.

**FIG 1:**
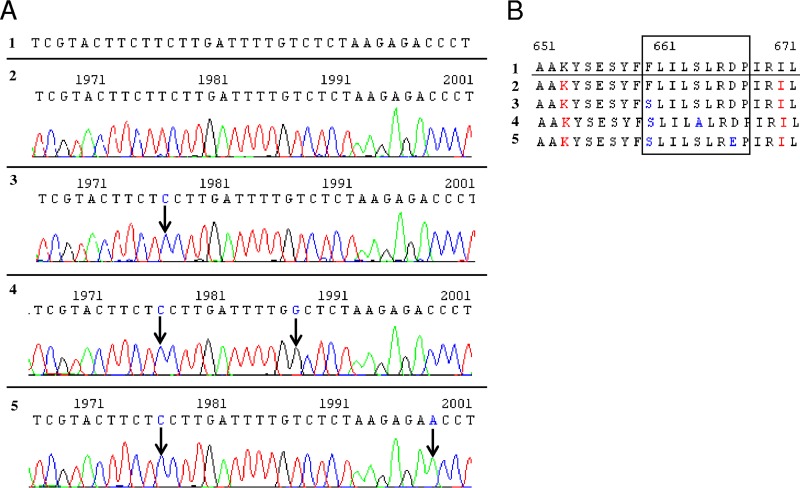
DNA sequencing chromatogram (A) and amino acid alignments (B) of the FKS2HS1 region in different C. glabrata isolates from patients. Lines 1, C. glabrata wild-type genome database sequence used for alignment (GenBank accession number XM_448401); lines 2, C. glabrata wild-type isolate; lines 3, F659S mutation found in resistant isolates from D12 to D61; lines 4, F659S and S663A mutations found in resistant isolates from D61 to D89; lines 5, F659S and D666E mutations found only in the D89 anus isolate. This isolate harbors another population with F659S and S663A mutations (lines 4).

For P1, *FKS2* HS1 mutations were detected in 22 resistant C. glabrata isolates. The first mutation, F659S, appeared after 14 days of treatment. The second mutation, S663A, appeared after 63 days of treatment, associated with the first. The third mutation, D666E, appeared once after 89 days of treatment (day 89 [D89] anus isolate) (Table 1). In this isolate, a mutated double population was observed, one population with F659S and S663A mutations and the other with F659S and D666E mutations (Fig. 1). For P2, one mutation in *FKS1* HS1 (S645P) was found in five C. dubliniensis isolates. For P3, the same *FKS* mutation in *FKS1* HS1 (S645P) was detected in one C. albicans isolate. For the three patients, no mutation was detected in the susceptible isolates for which *FKS* was sequenced (Table 1).

### Genotyping.

Susceptible and resistant isolates of C. glabrata, C. dubliniensis, and C. albicans harvested from P1, P2, and P3, respectively, all belonged to the same genotype. For C. glabrata, the sizes of the mitochondrial RNase P precursor gene (*RMP2*), the metallothionein I gene (*MTI*), and the 5,6-sterol desaturase (*ERG3*) gene microsatellites were 128, 242, and 228 bp, respectively. For C. albicans, the sizes of the 2 alleles of the elongation factor 3 gene (*EF3*), the cell division cycle protein gene (*CDC3*), and the imidazole glycerol phosphate dehydratase gene (*HIS*) microsatellites were 136 and 145, 116 and 128, and 152 and 152 bp, respectively. Multilocus sequence typing (MLST) of C. dubliniensis did not reveal any difference in polymorphism positions.

### Correlation between *FKS* mutations and MICs.

Using the EUCAST reference method, resistance was detected in 27/27 (100%) and 25/27 (93%) *FKS* mutant isolates for anidulafungin and micafungin, respectively. In contrast, the mutant isolates were detected as resistant or intermediate isolates by Etest in 15/27 (56%), 14/27 (52%), and 27/27 (100%) isolates for anidulafungin, micafungin, and caspofungin, respectively, when MIC values were interpreted with the CLSI clinical breakpoints (as recommended by the manufacturer). However, when the EUCAST breakpoints were used to interpret the MIC results, Etest detected resistance in 24/27 (89%) and 27/27 (100%) mutant isolates for anidulafungin and micafungin, respectively.

For C. glabrata, the first mutation, F659S, alone was associated with a low level of resistance (EUCAST MICs of 0.125 and 0.06 mg/liter for anidulafungin and micafungin, respectively), which already represented an increase of up to 4-fold compared with the *FKS* wild-type (WT) susceptible parent isolate. The resistance level increased with the second mutation (F659S and S663A), with MICs up to 32-fold higher than those of the WT. Finally, for a D89 anus isolate, the MICs were 32- to 512-fold higher than those of WT. Parts of the *MSH2* genes of four C. glabrata isolates (D−2 anus, D12 urine, D61 urine, and D89 anus) were sequenced, and no mutation was found.

## DISCUSSION

To our knowledge, this is the first study that has investigated echinocandin drug resistance in Candida spp. in a liver transplant recipient population. Our results showed that of the 39 patients treated with echinocandin, 3 (8%) acquired a resistant isolate. The same prevalence (8%) was reported for C. tropicalis in a hematology population where patients were treated with echinocandins (17). In our study, this prevalence may have been underestimated due to the use of Etest as a screening method rather than the EUCAST method; the latter seems more sensitive in detecting resistant isolates.

Genotyping revealed that, in the three patients concerned, resistant and susceptible isolates were isogenic. This confirms, as previously demonstrated in several studies (7, 19–22), that susceptible isolates acquired resistance under selection pressure in antifungal-treated patients. In our cases, the resistance developed rapidly, in less than 1 month. It appeared more rapidly in C. glabrata (14 days of echinocandin treatment) than in C. albicans (24 days) or C. dubliniensis (27 days with a preexposure period of 23 days, i.e., 51 days before). This rapid emergence of echinocandin resistance in C. glabrata has been described previously and is related to its haploid nature (19).

The positions of the first two mutations (Phe-659 and Ser-663) were previously reported in other echinocandin-resistant C. glabrata isolates (9, 23, 24). The substitution S663A and its association with F659S were described for the first time, in the same patient, by Garnaud et al. in a previous study on detection of resistance by next-generation sequencing (NGS) (25). The third mutation (D666E) is very rare and is found in less than 4% of resistant C. glabrata isolates (26). It was not detected by NGS in the Garnaud et al. study (25) because the sample collections stopped at D70, i.e., before the development of the third mutation. The mutation was found only in a D89 anus isolate and was associated with F659S (Fig. 1). This association (F659S and D666E) is described for the first time. It would be interesting to analyze the fitness cost of these mutations in an animal model and to test the kinetics of glucan synthase inhibition. Recently, the *MSH2* gene has been shown to promote echinocandin resistance (27). For this reason, we sequenced parts of the *MSH2* gene in four isolates recovered at different time points during echinocandin treatment, yet no mutation was detected in the gene. Our results clearly show the impact of echinocandin prophylactic treatment on the emergence of resistant C. glabrata spp. These findings are consistent with a recent study that suggested that colonizing mucosal flora may create a reservoir of resistant Candida spp., in particular for C. glabrata (20).

Our results show that during the appearance of the first two mutations in C. glabrata, the anidulafungin Etest was better than the micafungin Etest. The caspofungin Etest also showed good performance in detecting mutant isolates. Moreover, better performance of the Etest was obtained when EUCAST breakpoints were applied. For the EUCAST method, anidulafungin seems also to be the best echinocandin marker for detection of resistance, which is consistent with the use of anidulafungin as a marker for echinocandin susceptibility testing (28). However, it should be noted that our data are restricted to several isolates taken from one patient and that the present study was not designed to evaluate the performance of antifungal susceptibility testing methods.

The resistant C. dubliniensis isolates of P2 and the resistant C. albicans isolate of P3 harbored an S645P substitution in *FKS1* HS1, which is a mutation commonly found in C. albicans (12). Although the mutation has been reported for C. dubliniensis (29), the appearance of resistant isolates during echinocandin therapy is a phenomenon that has never been reported for the species. Most echinocandin-resistant isolates were sampled from or around digestive sites, like abdominal aspirates and drainage and peritoneal fluids. This is probably due to the low diffusion of echinocandin in these sites (30, 31), which leads to subinhibitory concentrations, thus promoting the emergence of resistance. In previous case reports, the caspofungin and micafungin concentrations in the bile and ascites fluid were 30% and 15% of their levels in serum, respectively (31, 32). Thus, as mentioned in a recent study, our results suggest that the abdomen could be the origin for the development of echinocandin-resistant Candida spp. (32). Echinocandins exhibit concentration-dependent effects on Candida spp. Preclinical pharmacokinetic and pharmacodynamic studies support the idea that the infrequent administration of large doses is a better strategy to achieve higher maximum concentrations of the drug in the serum (33). Moreover, once-weekly micafungin therapy seemed to be as efficacious as daily therapy in a murine model of disseminated candidiasis (34). Because this strategy cannot be applied in humans, it could be advisable to modify the antifungal treatment in cases of abdominal candidiasis in order to have better diffusion in abdominal sites.

In conclusion, our study sheds more light on the risk of emergence of resistance during echinocandin curative or prophylactic treatment, especially in digestive sites. These resistant isolates are not always associated with an infection but must be taken into account, since it has been shown that colonizing isolates are generally the same as those responsible for candidemia (35, 36). Accordingly, we are keen to raise awareness among clinicians and microbiologists of the emergence of echinocandin resistance, even during prophylactic treatment. Resistance can occur rapidly, which suggests that it is important to stop prophylaxis as soon as possible.

## MATERIALS AND METHODS

### Study design, patients, and isolates.

The study population included all the patients who received transplants in our LT center (Henri Mondor University Hospital, Créteil, France) between January and June 2013 and 2015. Data were collected prospectively from the patients' records. The study was approved by the local ethical committee, and the database was reported to the Commission Nationale de l'Informatique et des Libertés (CNIL) (no. 1699340).

The first phase of the study was directed at assessing echinocandin drug resistance and determining its location and its time of onset under antifungal pressure. This phase was conducted in patients who received echinocandin as curative or prophylactic treatment in compliance with the recommendations of Gavaldà et al. (3), because they were at high risk of IFI infection. The patients received caspofungin at 70 mg on day 1, followed by 50 mg/day, or micafungin at 100 mg/day. Patients who were at risk of IC received fluconazole at 400 mg/day. In cases of IC, patients were given a curative antifungal treatment with caspofungin or micafungin for at least 48 h, the time needed for the identification of the responsible Candida sp. and receipt of its susceptibility results.

All the patients were subjected to weekly monitoring of Candida colonization and were screened for Candida infections in blood cultures and in other sterile sites according to clinical signs. As part of this routine surveillance of Candida colonization, swabs were systematically taken from five superficial sites (mouth, nose, axillary surface, inguinal fold, and anus) on the day of admission to undergo LT and once per week thereafter until discharge from the intensive care unit (ICU) or death. Colonization was defined as the isolation of Candida species isolates from at least one surveillance site.

### Isolate identification and storage.

All clinical samples were cultured on Chromagar plates (Becton Dickinson) and incubated for at least 48 h at 37°C. Candida isolates were identified by matrix-assisted laser desorption ionization–time of flight (MALDI-TOF) (Microflex; Brucker) following standard extraction. The fungus species was identified using the MALDI BioTyper database version 3.0. For C. dubliniensis isolates, the species type was confirmed using *ITS1* and *ITS2* gene sequencing after mass spectrometry identification (37). All isolates were initially stored at −20°C on cryobeads (bioMérieux).

### Antifungal susceptibility testing.

Micafungin, anidulafungin, and caspofungin Etest strips (bioMérieux) were used to screen the susceptibility of all the Candida isolates from patients treated with echinocandin. When a resistant isolate was spotted, all the other isolates from that patient were subjected to Etest and EUCAST broth microdilution method testing. For Etest, the isolates were tested according to the manufacturer's instructions. A yeast suspension adjusted to 0.5 McFarland standard was used to inoculate RPMI 1640 agar plates (bioMérieux). Etest strips (bioMérieux) were then applied, and the plates were incubated for 48 h at 37°C. An 80% inhibition endpoint was applied for MIC determination, as recommended for echinocandins. For EUCAST (38), anidulafungin (Pfizer Pharmaceutical Group) and micafungin (Astellas Pharma, Inc.) were tested at concentrations ranging from 0.015 to 8 mg/liter in RPMI 1640 (Sigma-Aldrich) buffered to pH 7.0 with MOPS (morpholinepropanesulfonic acid) (Sigma-Aldrich) and supplemented with glucose to a final concentration of 2%. The inoculated plates were incubated for 24 h at 37°C. Caspofungin was not used, because neither of the two reference methods (CLSI and EUCAST) is currently recommended to test Candida susceptibility to caspofungin, due to problems in test reproducibility (39). MIC values were determined spectrophotometrically (Multiskan FC microplate photometer; Thermo Scientific) as the lowest drug concentration that resulted in inhibition of ≥50% of fungal growth in comparison with the growth in a drug-free control well. For both techniques (EUCAST and Etest), Candida parapsilosis ATCC 22019 and C. krusei ATCC 6258 were used as quality control strains. EUCAST MIC results were interpreted according to breakpoints published in the EUCAST breakpoint table v8.0 (http://www.eucast.org/clinical_breakpoints). As there are currently no Etest-specific breakpoints, Etest MIC values were interpreted according to CLSI breakpoints (40), as recommended by the manufacturer. For comparison, Etest MICs were also interpreted using the current EUCAST breakpoints. For C. dubliniensis, we tentatively used the same breakpoints as for C. albicans. Indeed, it has been shown that the wild-type upper limits of the MIC distribution for each of the echinocandins are identical in the two species (41).

### DNA extraction.

To extract DNA, the yeasts were first disrupted with MagNa Lyser Green beads (Roche Diagnostics) in a MagNa Lyser instrument (Roche). Then, proteinase K (Qiagen Sciences Inc.) was added, and the mixture was incubated for 1 h at 56°C. DNA was extracted using the QIAamp DNA blood minikit (Qiagen Sciences Inc.) following the manufacturer's instructions.

### PCR amplification and sequencing of HS regions within *FKS* genes.

We looked for mutations in HS regions within *FKS* genes in all EUCAST and Etest resistant isolates and in susceptible isolates that were recovered before and after the collection of a resistant isolate. The HS1 and HS2 regions of the *FKS1* and *FKS2* genes of C. glabrata and the HS1 and HS2 regions of the *FKS1* gene of C. albicans were sequenced as previously described (42, 43). The primers used for HS1 and HS2 of the *FKS1* gene of C. dubliniensis were designed based on GenBank accession number XM_002416855.1. The primers used for HS1 and HS2 regions of the *FKS1* gene of C. dubliniensis in this study were as follows: for *FKS1* HS1, 5′-TATTCTTTGCTGTCATGCCCTT-3′ and 5′-ACCCAAATAGAATGAACGACCA-3′; for *FKS1* HS2, 5′-AAGATTGGTGCYGGTATGGG-3′ and 5′-RGTDGCAAAACCTCTAGCAGT-3′.

Each sample reaction mixture contained 0.5 μM each primer, 1× PCR gold buffer (Applied Biosystems), 1.5 mM MgCl_2_ (Applied Biosystems), 2.5 mM deoxynucleoside triphosphate (dNTP) solution (Eurobio), 0.03 U of AmpliTaq Gold (Applied Biosystems), and RNase-free water up to a final reaction volume of 100 μl containing 20 ng of genomic DNA. Amplification was performed on a Mastercycler gradient (Eppendorf). The PCR conditions were initial denaturation at 95°C for 10 min, followed by 40 cycles of denaturation at 95°C for 30 s, annealing at 55°C for 30 s, and extension at 72°C for 1 min and a final extension at 72°C for 10 min. The amplicons were purified by passing through columns of the MinElute PCR purification kit (Qiagen Sciences Inc.), and both strands were sequenced by Sanger's method (Eurofins Scientific). Sequence alignments were analyzed using SeqScape v2.5 (Applied Biosystems) and compared with the genome database sequences (GenBank accession numbers XM_446406 and XM_448401 for *FKS1* and *FKS2* of C. glabrata, respectively; XM_716336.1 for *FKS1* of C. albicans; and XM_002416855.1 for *FKS1* of C. dubliniensis).

### PCR amplification and sequencing of the C. glabrata *MSH2* gene.

We looked for mutations in parts of the *MSH2* gene where the most predominant mutations were found (according to reference 27). The primers used in this study were as follows: *MSH2Fragment2*, 5′-TCACGTGGATTCAGCAGTT-3′, and *MSH2Fragment2*, 5′-TCGTTGCCTAATAGTTTTGCC-3′; *MSH2Fragment3*, 5′-TCGGTGGTTACCATAGTCCCTA-3′, and *MSH2Fragment3*, 5′-TCTGGGACCTTCAAAACTAAACTG-3′. The PCR mixture conditions and sequencing were the same as those described above. Sequence alignments were analyzed using SeqScape v2.5 (Applied Biosystems) and compared with the C. glabrata genome database sequences (GenBank accession number CR380955).

### Genotyping.

Genotyping was performed on all *FKS*-sequenced isolates. Genotyping of C. glabrata and C. albicans was performed as previously described (44, 45). Three microsatellites within *RMP2*, *MTI*, and *ERG3* were amplified for C. glabrata (44). Other groups of 3 microsatellites within *EF3*, *CDC3*, and *HIS* were amplified for C. albicans (45). Microsatellite PCR amplification was carried out using a GeneAmp 9700 thermal cycler (Applied Biosystems) in a 25-μl volume containing 20 ng of DNA. The composition of the PCR mixture was as follows: 0.5 μM each primer, 1× PCR buffer, 2.5 mM MgCl_2_, 0.2 mM each dNTP, and 1.25 U of AmpliTaq Gold. The PCR conditions were initial denaturation at 95°C for 8 min, followed by 30 cycles of denaturation at 95°C for 30 s, annealing at 60°C for 30 s, and extension at 72°C for 1 min and final extension at 72°C for 5 min. PCR products were diluted 1/100, 1/500, or 1/1,000 according to the intensities of PCR. They were added to HiDi formamide and 400HD size standard (Applied Biosystems) and denatured for 5 min at 95°C. They were then loaded on an ABI 3130 XL genetic analyzer (Applied Biosystems). The peak areas and the sizes of amplicons were determined using GeneScan analysis software v4.0 (Applied Biosystems).

Genotyping of C. dubliniensis was based on MLST profiles. Ten different loci were used for the MLST analyses: *AAT1a*, *AAT1b*, *ACC1*, *ADP1*, *GLN4*, *MPIb*, *RPN2*, *SYA1*, *VPS13*, and *ZWF1b* (46). PCR mixture conditions and sequencing were the same as those described above. Sequence alignments were analyzed using SeqScape v2.5 (Applied Biosystems).
